# How Epstein Barr virus shapes lupus autoimmunity: mechanistic insights and therapeutic perspectives

**DOI:** 10.3389/fimmu.2026.1853891

**Published:** 2026-06-02

**Authors:** Rada Miskovic, Ivica Jeremic, Danijela Miljanovic, Andja Cirkovic, Ana Banko

**Affiliations:** 1 Clinic of Allergy and Immunology, University Clinical Centre of Serbia, Belgrade, Serbia; 2 Faculty of Medicine, University of Belgrade, Belgrade, Serbia; 3 Imedic Polyclinic, Belgrade, Serbia; 4 Institute of Microbiology and Immunology, Faculty of Medicine, University of Belgrade, Belgrade, Serbia; 5 Institute for Medical Statistics and Informatics, Faculty of Medicine, University of Belgrade, Belgrade, Serbia

**Keywords:** autoimmunity, B-cells, Epstein–Barr virus (EBV), lupus pathogenesis, systemic lupus erythematosus (SLE), therapeutic implications

## Abstract

Systemic lupus erythematosus (SLE) is a chronic, complex autoimmune disease arising from the interaction of environmental triggers with a genetically susceptible host. Among environmental factors, infection with Epstein–Barr virus (EBV) has one of the strongest and most consistent epidemiological associations with SLE. Patients with SLE display higher EBV seropositivity rates, increased viral loads, and more frequent viral reactivations compared with healthy individuals. In genetically predisposed hosts, EBV can disturb immune homeostasis and contribute to the breakdown of self-tolerance, acting within a broader network of environmental influences that shape lupus pathogenesis. Despite this well-established association, the precise mechanisms linking EBV infection to lupus autoimmunity have remained incompletely understood. Recent single-cell RNA-sequencing revealed that EBV-infected B cells in SLE are transcriptionally distinct, with enhanced EBNA2-driven antigen presentation and ability to activate autoreactive CD4^+^ T cells, which positions EBV as an active driver rather than a passive trigger of autoimmunity. These findings also highlight the interconnections between EBV-related mechanisms and dominant lupus pathways. These important new insights may have significant therapeutic implications, with a focus on B-cell targeted therapies that may suppress EBV reservoirs, while T-cell approaches, vaccines, and therapies disrupting EBV latency programs emerge as possible new strategies. This Mini Review summarizes established and emerging mechanistic evidence connecting EBV to SLE, with particular emphasis on recent data identifying EBV-infected B cells as potentialdrivers of lupus pathogenesis, and discusses the therapeutic implications of these findings.

## Introduction

Systemic lupus erythematosus is a systemic autoimmune disease evolving from the interplay of genetic susceptibility and environmental triggers (1, 2). Epstein-Barr virus (EBV) is the most consistently implicated infectious factor (3, 4). It is a ubiquitous virus, also known as human herpesvirus 4 (HHV-4), with a worldwide prevalence exceeding 90% (5). It is transmitted predominantly through saliva. Primary infection usually occurs in early childhood and is typically asymptomatic, whereas in adolescence, it more frequently manifests as infectious mononucleosis (IM). Notably, IM and SLE share several clinical and laboratory features (6). Although epidemiological associations between EBV infection and SLE have been recognized for decades, the precise mechanistic link has long remained elusive. For a long time, this ambiguity left EBV positioned somewhere between a passive bystander and a possible trigger or modifier of lupus. However, accumulating recent evidence now indicates that EBV is not simply a passive factor, but an active participant in lupus pathogenesis, with the ability to amplify systemic autoimmunity (7, 8). This mini review explores current evidence linking EBV infection to SLE, focusing in particular on recent studies that have provided new mechanistic insights into this long-debated relationship. We highlight emerging data that position EBV-infected B cells as important drivers of immune dysregulation in lupus, and we discuss how these insights fit within existing models of SLE pathogenesis. Finally, we discuss the clinical relevance of these advances and their potential implications for novel therapeutic approaches in lupus.

## Overview of EBV biology

EBV is a double-stranded DNA herpesvirus that alternates between lytic replication and latency, with tightly regulated gene expression in each phase. Following transmission through saliva, EBV infects epithelial cells of the oropharynx. This initiates lytic replication, characterized by broad viral gene expression, genome replication, and release of new virions into saliva, facilitating further transmission (9). Subsequently, the virus enters B lymphocytes via binding of the viral glycoprotein gp350 to the CD21 receptor. This initiates lytic replication, characterized by broad viral gene expression, genome replication, and release of new virions into saliva, facilitating further transmission (9, 10). As immune control develops, EBV establishes latency in resting memory B cells, where the viral genome persists as an episome with highly restricted gene expression, thereby enabling immune system evasion. Periodic reactivation, potentially triggered by differentiation of infected B cells into plasma cells, restores lytic replication and promotes further viral spread (11).

The ability of EBV to establish lifelong persistence relies on multiple host immune system evasion strategies. During lytic infection, the virus expresses a viral interleukin-10 homolog, which suppresses interferon-γ (IFN-γ) production, attenuates CD8^+^ cytotoxic T-cell responses, and limits upregulation of MHC class I molecules, while another lytic protein - restricted early antigen (EA-R), acts as a viral counterpart of Bcl-2, protecting infected B cells and epithelial cells from apoptosis (9, 10). During latency, EBV promotes the survival of infected B cells by mimicking key B-cell survival signals: LMP-1 acts as a constitutively active CD40 analog, and LMP-2A reproduces key aspects of B-cell receptor signaling. Expression of latent viral genes is tightly controlled by EBNA-2, a central transcriptional regulator, while EBNA-1 is the only viral protein strictly required for viral persistence during latency, ensuring replication of the viral episome once per cell cycle (10). The glycine–alanine repeat domain of EBNA-1 limits proteasomal processing and MHC class I antigen presentation, allowing latently infected B cells to evade CD8^+^ T cell recognition (11).

## Epidemiological and serological evidence linking EBV to SLE

In SLE, the immune response to EBV is markedly dysregulated, resulting in frequent viral reactivations and increased replication. Several studies have reported markedly increased EBV loads in peripheral blood mononuclear cells of SLE patients, ranging from approximately 15-fold to as high as 40-fold above those observed in controls, independently of immunosuppressive therapy (12, 13). In contrast, detection rates of circulating cell-free EBV DNA are lower (14). A meta-analysis found EBV DNA positivity in 55.1% of SLE patients versus 20.7% of controls, corresponding to an odds ratio of approximately 3.9 (15). Impaired viral control appears to be related to quantitative and functional defects of EBV-specific CD8^+^ T cells, including reduced cytotoxicity and features consistent with T-cell exhaustion (16). Compensatory expansion of IFN-γ–producing EBV-specific CD4^+^ T cells has also been observed, together with reductions in Th17 and regulatory T-cell subsets (12, 17). Because general mechanisms of immune surveillance appear preserved, as evidenced by intact cytomegalovirus (CMV)-specific T-cell responses, this dysregulation is thought to reflect an intrinsic defect specifically affecting EBV immune control (6). Additional contributing mechanisms may include broader leukocyte dysfunction and altered cytokine responses to EBV antigens (18). Composite virological and serological markers further support a higher frequency of active or recent EBV infection in SLE patients than in controls. In one recent cohort, active EBV infection was observed in approximately 45% of patients (14). In addition, this study suggests that EBV infection is not only more frequent in SLE but may also be linked to its clinical phenotype and disease activity, further supporting its relevance in lupus pathobiology (19). Similar observations have also been reported in rheumatoid arthritis, where active or recent EBV infection was detected in 42% of patients, raising the possibility that impaired EBV control may extend beyond SLE to other systemic autoimmune diseases (20). As cellular immunity fails to control EBV infection, a compensatory shift toward humoral immunity occurs. A 2019 meta-analysis demonstrated significantly higher seropositivity for most anti-EBV antibodies in SLE patients compared to controls (15). Moreover, qualitative alterations in the humoral immune response have been reported (14, 19). More recent studies have further shown that markers of active or recent EBV infection, including anti-VCA IgM, anti-EA(D) IgG, and anti-EA(D) IgM, are more prevalent in SLE and often present at higher titers than in healthy individuals (19). Additionally, SLE patients more frequently produce IgA antibodies against EBV antigens, possibly due to the EBV reactivation in epithelial cells or reinfection of epithelial cells following reactivation in B cells (21). Some EBV antibodies could potentially serve as a marker of disease presence. Among EBV-related serological markers, anti-EBV EA(D) IgG may be of particular interest, as its presence was independently associated with SLE in a recent study and corresponded to an approximately 24-fold higher odds of disease compared with controls, possibly reflecting persistent immune responses to lytically infected cells expressing EA(D) (19). The only stronger serology-based association reported to date is the 32-fold increased risk of multiple sclerosis following EBV infection (22). Beyond disease association, anti-EBV EA(D) IgG antibodies have also been linked to specific clinical phenotypes, particularly hematologic and renal involvement, in line with prior reports connecting anti-EA responses with heightened IFN-driven inflammation and renal manifestations (23, 24). EBV antibody profiles may also hold potential predictive value in selected clinical settings. In a prospective study, anti-EA(D) IgM antibodies were associated with achievement of remission and low disease activity, particularly in patients with mucocutaneous involvement (14).

Further epidemiological evidence supporting the link between EBV and SLE comes from prospective studies in individuals at increased risk of disease. In a 2019 longitudinal study of 463 unaffected relatives of SLE patients followed for 6.3 years found that 13% developed SLE. Although EBV seroprevalence was similar, individuals who transitioned to disease had higher baseline anti-VCA IgG and anti-EA(D) IgG levels, which, together with identified gene–environment interactions involving CD40 and IL-10 variants, suggested that impaired genetic control of latent EBV infection may predispose to recurrent viral reactivation and increased risk of SLE (25).

## EBV mimicry and epitope spreading in SLE

Molecular mimicry, together with epitope spreading, represents one of the key mechanisms by which EBV may contribute to lupus autoimmunity. Several EBNA-1 regions share cross-reactive epitopes with major lupus-associated autoantigens, including Ro, dsDNA, SmB, SmD, and C1q (26–28). Notably, a proline-rich peptide sequence of the small nuclear ribonucleoprotein (snRNP) has been identified as an initial target in autoimmune responses. Immunization of experimental animals with a cross-reactive EBNA-1 derived epitope can induce the production of anti-Ro antibodies and lupus-like manifestations. In this context, epitope spreading may expose additional cryptic nuclear epitopes, including determinants within RNP1, U1-RNP, and RNP-C related antigens, thereby broadening the autoantibody repertoire and ultimately contributing to the transition to clinically manifest SLE (28, 29). Autoreactive B cells play a central role in this process, acting not only as producers of autoantibodies but also as antigen-presenting cells (APCs) that sustain pathogenic T cell responses. These cells diversify within distinct anatomical niches, including extrafollicular foci and germinal centers in secondary lymphoid organs, while tertiary lymphoid structures offer tissue-specific *in situ* diversification. Epitope spreading may also contribute to therapy resistance in lupus patients by maintaining diversification of autoreactive B−cell responses despite depletion of circulating CD20^+^ B cells (29).

Peptides derived from other EBV antigens, including EA, LMP1, and LMP2A, have also been implicated in the induction of an autoimmune response. In an animal model, immunization with peptides derived from these viral antigens led to increased ANA, anti-SmB, and anti-SmE autoantibodies, supporting the concept that multiple EBV proteins may contribute to the diversification of autoimmunity (30). More recent findings have also highlighted a potential role of another latent viral protein, EBNA2, in the pathogenesis of autoimmunity. Genome-wide analyses have shown that EBNA2 occupies nearly half of SLE risk loci, with additional evidence of allele-dependent binding and downstream effects on gene expression, supporting a model in which this viral transactivator intersects with host susceptibility pathways to shape disease-relevant regulatory programs. This type of association has also been found in multiple sclerosis, rheumatoid arthritis, type 1 diabetes, celiac disease, and inflammatory bowel disease (31).

Several viral proteins mediate functional mimicry, facilitating immune evasion and enhancing autoimmune response. In lupus-prone experimental models, LMP1, an EBV-encoded mimic of CD40 signaling, amplifies autoimmunity by promoting B-cell activation and costimulation, enhancing T-cell activation, and thereby sustaining pathogenic B-cell responses (32). Additionally, in a more recent study, increased expression of the LMP1 gene has been reported in SLE patients and linked to both disease activity and the interferon pathway activation, further supporting its potential relevance to human disease (33). If validated in further studies, LMP1-related pathways could represent potential therapeutic targets in SLE.

During lytic infection, the EBV-encoded viral interleukin-10 homologue contributes to immune evasion by modulating cytokine production and attenuating antiviral cellular immunity, thereby favoring B-cell survival, viral persistence, and reactivation, contributing to autoimmunity (6). It is plausible that an interplay of multiple EBV proteins contributes to the development of SLE, as shown in animal models, highlighting the importance of further clinical and epidemiological studies to translate findings to human disease (34). Additionally, within the framework of EBV-driven molecular mimicry, the impact of viral genetic variability on systemic autoimmunity remains poorly understood. It is conceivable that specific EBV variants, particularly within highly immunogenic regions such as EBNA1, may differentially shape cross-reactive immune responses and possibly influence clinical phenotypes. However, direct evidence in SLE remains limited, and this question warrants further investigation (19, 35, 36).

## New insights: EBV-mediated reprogramming of autoreactive B cells

Recent work by Younis et al. offered a conceptual shift regarding the role of EBV in lupus pathogenesis, showing that EBV-infected autoreactive B cells are active drivers of systemic autoimmunity. Using RNA-sequencing-based approaches, the study revealed transcriptional differences between infected and uninfected B cells in SLE patients, and showed that viral EBNA2 protein is the key driver (8). It showed that EBV infects antinuclear antigen-reactive B cells in SLE patients, whereas such autoreactive EBV^+^ B cells were not detected in healthy controls. In SLE, EBV^+^ B cells were predominantly CD27^+^CD21^low^ memory B cells expressing ZEB2 and TBX21, and trajectory analyses suggested that these cells arise from EBV^+^ naive, follicular, and double-negative, B-cell population (37). Mechanistically, the authors also demonstrated EBNA2 binding at regulatory regions of ZEB2, TBX21, and APC genes, providing a molecular basis for the acquisition of APC-like function by these B cells. This indicates that EBV has ‘‘hijacked’’ a transcriptional program that reprograms autoreactive B cells into persistent, antigen−presenting, pro−inflammatory effector cells that have a significant role in SLE pathogenesis. The finding was further supported by the finding demonstrating that EBV^+^ B cells from patients with SLE could present chromatin antigens and promote ANA responses, whereas EBV^+^ B cells from healthy controls lacked this capacity. Considering that low-level self-reactivity is necessary to support normal B-cell maturation, this could explain the apparent selectivity for SLE, whereby EBV preferentially targets pre-existing autoreactive naive B cells in genetically susceptible hosts and reprograms them into driver cells for systemic autoimmunity (8, 29). In this context, EBNA2 emerges as a potential therapeutic target. Further research should determine whether this EBNA2−driven reprogramming of autoreactive B cells is also relevant in other EBV−associated autoimmune diseases.

Interestingly, the study by Younis et al. also provides new insight into the long-standing question of whether EBV reactivation, which has been associated with lupus onset and disease flares, is a driver of these events or instead occurs as a consequence of immune activation (8, 14, 19, 23, 38). The authors detected small populations of EBV^+^ naive B cells, memory B cells, DN1 and DN2 B cells, and plasmablasts expressing lytic EBV genes in SLE patients, but not in healthy controls. This finding suggests ongoing viral reactivation that may involve abortive and overt lytic cycles. Considering that differentiation of memory B cells into plasmablasts induces transcription factors, which can also activate EBV lytic gene BZLF1, some of the observed lytic EBV gene expression may reflect a secondary consequence of B-cell activation rather than the primary driver of disease (8). These findings provide a useful framework for future research aimed at better clarifying the temporal relationship between EBV reactivation and the onset or flares of lupus.

## Positioning EBV within SLE pathogenesis frameworks

New evidence positions EBV as an active mechanistic contributor to SLE pathogenesis, and also provides a basis for why only a fraction of EBV infected individuals develop SLE. How do these new EBV data fit into current models of SLE pathogenesis?

Classic SLE models focus on a breakdown of immune tolerance, triggering activation of autoreactive B- and T-cells, interferon-mediated inflammation, epitope spreading, and chronic immune stimulation. Current findings regarding EBV infection refine these frameworks by highlighting that EBV preferentially infects autoreactive antinuclear antigen-reactive naive B cells, present in elevated numbers in SLE patients, positioning these cells as a potential link between B-cell autoreactivity, T-cell help, and interferon signaling (8). Many factors drive their expansion, including impaired anti-EBV cytotoxic T cell responses, dysfunctional regulatory T cells, EBV-mediated immune evasion, therapeutic immunosuppression, and other contributing factors (12, 16–18). EBV reprograms these infected autoreactive naive B cells into activated APCs (CD27^+^CD21^low^ZEB2^+^ T-bet^+^) through EBNA2-mediated transcriptional and epigenetic remodeling, making them capable of directly activating autoreactive CD4^+^ T-cells, amplifying further the autoimmune cascade. These CD4^+^ T-cells may also activate EBV^–^ B-cells and other autoreactive B-cells that recognize similar autoantigens, providing them with signals to transform into plasmablasts capable of secreting autoantibodies of different specificities, expanding significantly the autoantibody repertoire (7, 8).

In this way, EBV intersects with multiple SLE pathways. EBV-driven activation of infected B- and T-cells can promote IFN-α production, with serologic markers of EBV reactivation correlating with elevated IFN associated molecules and higher disease activity in IFN-high SLE subsets (23, 24). EBV-infected autoreactive B cells that acquire an APC-like phenotype may sustain extrafollicular B-cell responses and plasmablast expansion, intersecting with B-cell-driven tissue damage and autoantibody production (8, 39). Structural mimicry between EBV-derived antigens and self-proteins contributes to the broadening of the autoantibody repertoire and epitope spreading (26, 27). EBV-driven B-cell activation and antigen presentation may also lower the threshold for autoreactive CD4^+^ T-cell activation and skew T-helper responses, thereby reinforcing the aberrant T-B collaboration central to SLE pathogenesis (39).

**Figure 1:**
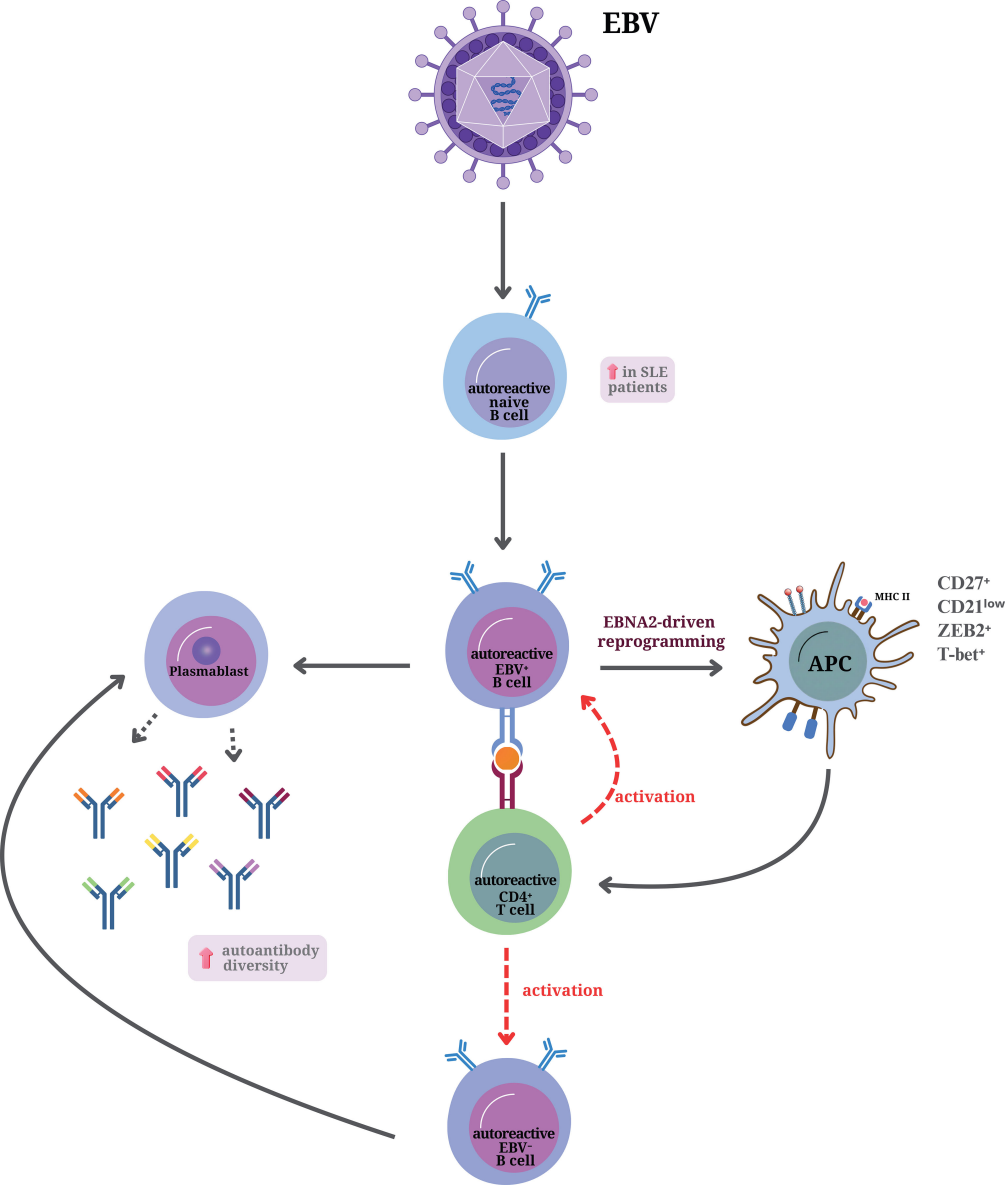
Proposed model of EBV- driven pathogenesis of systemic lupus erythematosus.

Additionally, the pathogenic contribution of EBV may be particularly relevant in selected SLE subsets, with stronger mechanistic links in SLE subsets characterized by high IFN activity and prominent B-cell dysregulation. In lupus nephritis, EBV-driven B-cell hyperactivation may preferentially amplify anti-dsDNA and anti-C1q antibody production relevant to immune complex deposition and renal flares in a subset of patients (40). In neuropsychiatric SLE, intrathecal EBV reactivation has been proposed to contribute to local neuroimmune inflammation, though direct evidence remains limited (41, 42). Overall, EBV infection dominantly influences B-cell-mediated autoimmunity while also mechanistically explaining the chronic, relapsing-remitting course of SLE through persistent latent infection and periodic reactivations sustaining B-cell stimulation, and thereby driving key inflammatory pathways. (**Figure 1**).

## Therapeutic implications and future directions

These novel mechanistic insights into the role of EBV in SLE pathogenesis point to several promising therapeutic directions. First, they reinforce the rationale B-cell-targeted therapies, including anti-CD20 mediated depletion and BAFF-directed strategies, which may reduce autoreactive B-cell activity and, at least indirectly, diminish EBV-harboring B-cell reservoirs. In this context, established B-cell-directed therapies such as rituximab (used off-label in selected refractory cases) and belimumab, as well as newer agents such as obinutuzumab - a type II anti-CD20 antibody now approved for active lupus nephritis and supported by recent positive phase 3 data in active SLE, reinforce the therapeutic relevance of targeting pathogenic B-cell compartments; by contrast, ianalumab remains an investigational BAFF-R targeted therapy currently being evaluated in phase 3 studies (43, 44). Earlier anti-CD22 targeting with epratuzumab and anti-BAFF strategies such as tabalumab failed in phase 3 trials, while atacicept, a dual BAFF/APRIL blocker, was halted prematurely due to severe hypogammaglobulinemia and serious infections, illustrating that both lack of efficacy and toxicity can derail mechanistically plausible approaches. By 2020, approximately 40 SLE clinical trials had failed, largely due to disease heterogeneity and insufficient patient stratification. These challenges equally apply to EBV-directed strategies and underscore the need for biomarker-driven patient selection, potentially by EBV-driven disease subsets, in future trial design (45–47). However, even effective B-cell depletion may not fully eliminate tissue-resident B cells and long-lived plasma cells, and it does not directly target the viral programs that may sustain autoreactive immune circuits (48). Importantly, EBV-driven epitope spreading, responsible for expanding autoreactive repertoires despite partial B-cell targeting, emerges as a driver of therapy resistance, as persistent plasma cells and tertiary lymphoid structures sustain diversification and relapse (29). Therefore, deep B-cell depletion strategies like CD19-CAR T-cell therapy may hold promise, especially for EBV-driven subsets characterized by EBV^+^ B-cell signatures. However, CAR-T approaches carry substantial risks, including cytokine release syndrome, neurotoxicity, and prolonged immunosuppression, and their application in SLE remains at an early investigational stage requiring careful patient selection and long-term safety monitoring. In this context, a recent (preprint) study indicated that 9G4id^+^ B cells, which are intrinsically autoreactivity-prone, may be a useful target in SLE (49, 50). Given that EBV favors expansion of autoreactive B cells, these findings may support the rationale for 9G4-targeted CAR/cTCR approaches that could simultaneously deplete the viral reservoir and pathogenic autoreactive clones. Another approach for consideration would be selective depletion targeting only EBV^+^ B cells (e.g., via EBV-specific CAR-T). Whether these approaches could achieve comparable efficacy with reduced toxicity warrants further investigation (8, 51, 52).

Disrupting EBV latency programs and EBNA2−driven transcription also holds promise as a potential mechanism−based approach. Targeting EBNA1 could disrupt EBV immune evasion by blocking critical host-virus interactions, and represents a promising preclinical target, though without *in vivo* validation so far (53). EBNA2-targeted TAT (Trans-Activator of Transcription) peptides have been demonstrated to inhibit EBV-driven B-cell proliferation *in vitro*, suggesting potential to block infected B-cell transformation, which is central to SLE pathogenesis (54). Given the central roles of EBNA1 and EBNA2 in molecular mimicry and autoreactive B-cell reprogramming, selective dual targeting could potentially halt early autoimmune cascades while sparing non-infected B cells. LMP1, which is upregulated in SLE flares and promotes B-cell activation and T-cell help, may also be a potential therapeutic target. Although derived primarily from cancer studies, LMP1-derived vaccines elicited strong anti-EBV T-cell responses (mouse models) and LMP2-specific cytotoxic T lymphocytes (currently in clinical trial), together suggesting that EBV-directed immunotherapies may eventually hold relevance for selected SLE subsets (55, 56). It should be noted that all EBNA- and LMP-targeting strategies remain at preclinical or early experimental stages, and off-target effects on non-infected B cells, as well as potential immune dysregulation from disrupting latency programs, remain important unresolved safety concerns.

EBV vaccination holds promise for primary prevention in genetically susceptible individuals. However, development is complicated by significant mimicry between viral and human-derived peptides, the same molecular mimicry implicated in SLE pathogenesis, which raises concerns that vaccine-induced immune responses could paradoxically trigger or exacerbate autoimmunity in genetically susceptible individuals, a risk that must be carefully addressed in trial designs. Considering strong epidemiological evidence linking the timing of EBV infection to later SLE risk, early-life vaccination represents a plausible preventive approach that could possibly interrupt disease-relevant pathways before autoimmune cascades are established. Several prophylactic EBV vaccine candidates, including mRNA-1189, gp350-Ferritin nanoparticle platforms, are currently being evaluated in early-phase clinical trials primarily for safety, immunogenicity, and prevention of EBV infectious mononucleosis. If successful, such strategies could conceivably reduce the future incidence of EBV-associated autoimmune diseases, including SLE, in genetically susceptible populations (NCT04645147) (57). Therapeutic gene-silencing approaches also warrant consideration. In particular, antisense oligonucleotides targeting EBV transcripts such as EBNA1 and LMP1 offer additional promise as a potential therapeutic strategy aimed at disrupting viral persistence and immune evasion, conceptually analogous to antisense therapy previously approved for CMV retinitis (58). An overview of potential therapeutic strategies based on EBV-related mechanisms in SLE pathogenesis is summarized in **Table 1**.

**Table 1:** Summary of potential therapeutic strategies based on the role of EBV in SLE.

Therapeutic approach	Mechanistic rationale/therapeutic target	Potential benefits	Limitations
Conventional B-cell targeting	Broad B-cell depletion and BAFF pathway inhibition reduce autoreactive B cells and EBV reservoirs	Decreases autoimmune B-cell activity; partial suppression of EBV reservoir	Incomplete depletion (tissue-resident B cells); does not directly target EBV (48)
Deep B-cell depletion with CD19-CAR T-cell therapy	Eliminates EBV-infected and autoreactive B-cell compartments	Potential eradication of EBV^+^ B-cell reservoirs	High toxicity risk; long-term safety and durability unclear (51, 52)
Precision autoreactive B-cell targeting (9G4)	Selective targeting of 9G4id^+^ (autoreactivity-prone) B cells enriched in SLE and potentially expanded by EBV	Removes key pathogenic clones; may reduce both autoantibodies and EBV reservoir	Early-stage evidence; not yet clinically validated (49)
EBV latency disruption	Targeting EBV transcriptional programs (e.g., EBNA1, EBNA2) to block viral persistence	Interrupts upstream disease drivers	Mostly preclinical; limited *in vivo* validation (53, 54)
Viral protein–targeted immunotherapy	Immune targeting of EBV latent proteins (e.g., LMP1, LMP2) to enhance antiviral T-cell responses	Strengthens anti-EBV immunity and reduces pathogenic B-cell activation	Evidence mainly from oncology and early-phase studies (55, 56)
Antisense therapy	Silencing EBV transcripts (e.g., EBNA1, LMP1) using oligonucleotides	Direct inhibition of viral gene expression; targeted approach	Experimental; not yet clinically established (58)
EBV vaccination	Prevention of EBV infection and establishment of latent reservoir in genetically susceptible individuals	Potential primary prevention of SLE and other EBV-driven diseases	Risk of molecular mimicry; early clinical development (57)

BAFF, B cell activating factor; EBV, Epstein-Barr Virus; CAR-T cell, Chimeric Antigen Receptor T cell; EBNA, Epstein–Barr Nuclear Antigen; LMP, Latent Membrane Protein.

## Conclusion

A growing body of evidence supports a role for EBV in SLE across the disease continuum, from early immune dysregulation to flares, clinical expression, and treatment responses. EBV is increasingly recognized not as a passive bystander, but as an active and persistent contributor to lupus autoimmunity. Similar mechanisms are likely relevant in other autoimmune diseases, including multiple sclerosis, Sjögren’s disease, and rheumatoid arthritis. Taken together, these advances support a shift toward more integrated mechanistic and translation research, with the aim of identifying robust biomarkers and developing precise, mechanism-based therapies that could meaningfully improve the prevention, stratification, and treatment of autoimmune disease.
